# Optical Mapping of Brain Activity Underlying Directionality and Its Modulation by Expertise in Mandarin/English Interpreting

**DOI:** 10.3389/fnhum.2021.649578

**Published:** 2021-08-06

**Authors:** Yan He, Yinying Hu, Yaxi Yang, Defeng Li, Yi Hu

**Affiliations:** ^1^College of Foreign Languages and Literatures, Fudan University, Shanghai, China; ^2^School of Psychology and Cognitive Science, East China Normal University, Shanghai, China; ^3^Centre for Studies of Translation, Interpreting and Cognition, University of Macau, Macau SAR, China

**Keywords:** interpreting directionality, interpreting expertise, fNIRS, right Broca’s area, right dorsolateral prefrontal cortex, right superior temporal gyrus

## Abstract

Recent neuroimaging research has suggested that unequal cognitive efforts exist between interpreting from language 1 (L1) to language 2 (L2) compared with interpreting from L2 to L1. However, the neural substrates that underlie this directionality effect are not yet well understood. Whether directionality is modulated by interpreting expertise also remains unknown. In this study, we recruited two groups of Mandarin (L1)/English (L2) bilingual speakers with varying levels of interpreting expertise and asked them to perform interpreting and reading tasks. Functional near-infrared spectroscopy (fNIRS) was used to collect cortical brain data for participants during each task, using 68 channels that covered the prefrontal cortex and the bilateral perisylvian regions. The interpreting-related neuroimaging data was normalized by using both L1 and L2 reading tasks, to control the function of reading and vocalization respectively. Our findings revealed the directionality effect in both groups, with forward interpreting (from L1 to L2) produced more pronounced brain activity, when normalized for reading. We also found that directionality was modulated by interpreting expertise in both normalizations. For the group with relatively high expertise, the activated brain regions included the right Broca’s area and the left premotor and supplementary motor cortex; whereas for the group with relatively low expertise, the activated brain areas covered the superior temporal gyrus, the dorsolateral prefrontal cortex (DLPFC), the Broca’s area, and visual area 3 in the right hemisphere. These findings indicated that interpreting expertise modulated brain activation, possibly because of more developed cognitive skills associated with executive functions in experienced interpreters.

## Introduction

As interlingual communication is growing rapidly, bilingual speakers interpreting from one language into another is becoming a widespread phenomenon. In interpreting practice, the issue of directionality has attracted wide attention (i.e., Klein et al., 1995; Price et al., 1999; García et al., 2016; Jost et al., 2018). Usually, it refers to the unequal cognitive cost between interpreting from L1 (the mother tongue) to L2 (the second language; forward interpreting, FI) and from L2 to L1 (backward interpreting, BI). In spite of the wide attention it received, the neural mechanisms underlying directionality are not well-understood yet.

According to the *inhibitory control* model (Green, 1998), when bilingual speakers translate from L1 to L2, inhibition of L1 words is required so as to produce L2 words, and vise versa. Inhibiting the active words in the non-target language takes time and yields a cost in cognitive effort (Price et al., 1999). Two variables have been identified that can affect the amount of inhibition: the level of activation associated with the words that must be inhibited and the speaker’s proficiency level in the non-target language (Green, 1986). Based on this model, L1 lemmas are more active than L2 lemmas in the brain of unbalanced bilinguals. Therefore, translation from L1 to L2 requires more cognitive effort to suppress L1 lemmas, compared to translation from L2 to L1 which suppresses L2 lemmas.

Interestingly, neuroimaging studies have been performed to examine the neural substrates of the directionality effect in translation and interpreting by using various techniques, such as positron emission tomography (PET), electroencephalography (EEG), and functional near-infrared spectroscopy (fNIRS) although no congruent results have been obtained in early neuroimaging studies (Klein et al., 1995; Kurz, 1995; Price et al., 1999; Rinne et al., 2000; Quaresima et al., 2002). Basically, the studies were focused on examining whether the directionality effect exists, and identifying the brain regions that are correlated with the translation/interpreting process. For example, Klein et al. (1995) discovered in a PET study that both the forward and backward translation was correlated with the significant neural activity in inferior and dorsolateral frontal and prefrontal regions. In particular, compared to that of the backward translation, increased neural activity occurred in the left putamen known to be involved in the forward translation. Rinne et al. (2000) reported a similar directionality effect, in which they discovered that compared to shadowing, both the forward and backward translation tasks resulted in a strong increase in activity within the left frontal lobe, including the dorsolateral frontal cortex. More importantly, significantly enhanced brain activity was observed in Broca’s area that controls the forward translation. In an EEG study, Kurz (1995) also demonstrated the directionality effect. By contrast, they found that relative to resting state neural activity, interpreting tasks produced more pronounced brain activation in the left temporal cortex and the right hemisphere involved more in the forward translation. Recently, Jost et al. (2018) concluded, in their electroencephalography (EEG) study, that translating from L1 to L2 involved greater activation in brain regions associated with attention, arousal, and awareness. In addition, García et al. (2016) and Zheng et al. (2020) found different connectivity patterns between the two translation directions. However, this was not the case for the study conducted by Price et al. (1999) and Quaresima et al. (2002), in which the translation asymmetry effect was not revealed by neuroimaging techniques. To date, despite growing neuroimaging evidences in translation/interpreting process, there is no systematic neural evidence for specific differences between the forward and backward translation.

Also, this area of research has been understudied regarding the impacts of translation/interpreting expertise on directionality. Expertise refers to the mastery of outstanding skills that has been obtained through years of deliberate practice and experience (Ericsson et al., 2007). The results obtained from empirical studies have indicated that the development of interpreting skills may enhance specific executive functions in bilinguals (e.g., Yudes et al., 2011; García, 2014). However, as suggested by García (2014), more studies remain necessary to determine whether the advantages obtained by bilinguals with interpreting expertise compared with non-interpreting bilinguals are similar in both L1 and L2 tasks. For example, in a study by Christoffels et al. (2006), professional interpreters performed similarly in terms of the accuracy of their interpreting during speaking and reading span tasks in L1 and L2. Conversely, their performances were incongruent in other tasks, such as semantic error detection (Fabbro et al., 1991) and word span (Christoffels et al., 2006). No clear patterns have emerged from these preliminary data or studies comparing student interpreters with non-interpreter bilinguals (Chincotta and Underwood, 1998; Tzou et al., 2011). Also, little research has been conducted concerning how directionality is affected by task difficulty. In the present study, we also included tasks of different difficulty to investigate this issue.

Previous studies have implicated the prefrontal cortex, especially the dorsolateral prefrontal cortex (DLPFC), in executive control, such as working memory, cognitive flexibility, planning, inhibition, and abstract reasoning (Miller and Cummings, 2007). Broca’s area has been associated with various language-related functions, including verbal working memory (Kovelman et al., 2008), morphosyntactic processing (Laine et al., 1999), and semantic analysis (Cabeza and Nyberg, 1997). The left/right superior temporal gyrus has been linked to semantic and syntactic processing (Friederici et al., 2003), and the left/right inferior parietal lobule has been associated with language comprehension (Ramachandran and Hubbard, 2003; Hartwigsen et al., 2010) and high-order language activities (Brownsett and Wise, 2010). Above all, a neural association has been suggested between interpreting and the prefrontal cortex (Klein et al., 1995; Ren et al., 2019), Broca’s area (Tommola et al., 2000; He et al., 2017; Shinozuka et al., 2021), the superior temporal gyrus (Hervais-Adelman et al., 2017; Shinozuka et al., 2021), and inferior parietal lobule (Price et al., 1999). By focusing on these brain areas, this study aimed to identify the neural activation patterns associated with directionality in interpreting.

Based on the *inhibitory control* model and previous neuroimaging studies, two hypotheses were tested. The first hypothesis was that a directionality effect exists, such that FI elicits more pronounced brain activity in bilinguals who are more proficient in L1 than in L2. The second hypothesis was that directionality is modulated by interpreting expertise. fNIRS, which is an increasingly popular, non-invasive, neuroimaging technique, was utilized in the present study. Compared with PET and functional magnetic resonance imaging (fMRI), fNIRS offers unsurpassed temporal resolution and provides quantitative hemodynamic information regarding oxyhemoglobin (HbO) and deoxyhemoglobin (HbR; Yuan, 2013). fNIRS has been shown to be relatively insensitive to movement artifacts, allowing study designs to include body movements; this is especially helpful because the present study requires that participants engage in continuous overt speech. The effects of interpreting expertise on brain activation patterns have been scarcely explored in the context of sight translation (one modality in interpreting) between English and Mandarin; therefore, this pilot study provides a new avenue for better understanding the neural mechanisms underlying sight translation.

## Materials and Methods

### Participants

Two groups of bilingual postgraduate students were recruited through an online subject-recruiting forum/WeChat group. One group consisted of interpreting majors, recruited from Shanghai International Studies University, which was referred to as the interpreting group (IG), and the other group consisted of non-interpreting students from East China Normal University, referred to as the non-interpreting group (NIG). This type of division among participants has been extensively used in previous translation/interpreting studies (Chincotta and Underwood, 1998; Christoffels et al., 2003; Tzou et al., 2011). All of the recruited students were native Mandarin (L1) speakers, with English (L2) as their foreign language. A total of 28 interpreting students and 32 non-interpreting students were recruited as potential participants for this experiment.

Before the experiment, thorough screening was performed to ensure that the two groups were as comparable as possible, in terms of age, education, L2 proficiency, and language-history data, except for interpreting experience. The participants were screened by employing subtests (Reading Comprehension and Structure and Written Expression) from TOEFL as L2 proficiency test, which is suggested by Hulstijn (2010). Each potential participant was also asked to complete the Mandarin version of a working memory span test (Zhang, 2011), as studies have suggested the existence of a strong correlation between the executive function and working memory span (Padilla et al., 2005; Ibanez et al., 2010; Yudes et al., 2011). The final IG included 16 interpreting students (14 females and two males; mean age: 24.94 years, standard *deviation*_(*SD*)_ = 2.35), 19% of them were early bilinguals; whereas the final NIG consisted of 16 non-interpreting students (13 females and three males; mean age: 23.69 years, *SD* = 1.03), 25% of them were early bilinguals. The two groups were matched for a variety of language-related factors and working memory span but differentiated by interpreting experience (see Table 1 for detailed results). Among these factors, the percentage of L2 usage per week was calculated by dividing the total hours of using English per week, including reading, writing, speaking, and listening, into the number of total hours of a week. And the total time length of exposure to L2 was the sum of the total time length of living in a foreign country where people are native English speakers. Although the members of the IG were interpreting postgraduates, they had 1 year of professional interpreting experience on average; thus, they are considered qualified as professional interpreters at a junior level.

**Table 1:** Characteristics of participants in the study.

Item	IG			NIG		
	Mean	*SD*	Range	Mean	*SD*	Range
Age (years)	24.94	2.35	23–33	23.69	1.03	22–26
L2 proficiency score (max:100)	88.75	8.38	73–100	86.31	6.84	76–100
Age of first acquisition of L2 (years)	10.25	2.11	6–14	8.75	2.89	3–13
Working memory span amplitude (max:6)	2.75	0.45	2–3	2.81	0.66	2–4
Interpreting experience (months)**	14.29	15.91	1–60	1.35	2.98	0–12
Percentage of L2 usage per week (%)	37.44	0.21	3–90	24.13	0.18	4–40
Total time length of exposure to L2 (months)	0.28	0.77	0–3	1.50	3.08	0–10
Total time length since L2 acquisition (years)	14.63	2.94	10–23	14.75	2.49	10–20

**p < 0.01.

The experiment was done in the lab at the School of Psychology and Cognitive Science, East China Normal University. All participants were in a healthy condition during the experimental period. No individuals with reported histories of medical illness, neurological or psychiatric disorders were included in this study. All subjects were right-handed, as assessed by the Edinburgh Handedness Inventory (Oldfield, 1971), with normal or corrected-to-normal vision. All participants signed informed consent forms prior to the experiment and were paid for their participation. The protocol was approved by the Committee on Human Research Protection of East China Normal University (HR 094-2018).

### Materials

The study used sentences (in both Mandarin and English) as stimuli, and the feasibility and reliability of sentence translation for the purposes of neuroimaging research have been demonstrated by a substantial number of studies (e.g., Lehtonen et al., 2005; Scherer et al., 2012; Hervais-Adelman et al., 2015). The stimulus package contained 48 Mandarin and 48 English sentences. The sentences were constructed with exactly the same structure, in both languages, which was “subject + verb + object + complement”. To guarantee the consistency and clarity of the structure, infinitive phrases were used as the complement. Predicate verbs, which could be followed by both an object and a complement, were also employed. Therefore, the general structure of each sentence was “I/he/she/you/we/they + verb + [somebody] + to do [something]” in Mandarin and English versions. To allow for data comparisons across languages, the word count, word frequency, notional word density, pronoun density, and translatability (the difficulty of translating a sentence, as rated by five interpreting teachers) of each sentence were controlled (see Supplementary Material Appendix 1). Then, both the Mandarin and English sentences were divided evenly according to the difficulty level, based on average word frequency (Jensen, 2009) and translatability. The sentences were divided into two subsets: low-complexity (LC) and high-complexity (HC) sentences (see Supplementary Material Appendix 2). By including low- and HC sentences, we also investigated how directionality was affected by task difficulty.

### Procedure

The experiment consisted of four tasks: sight translation from Mandarin to English (FI), sight translation from English to Mandarin (BI), reading aloud in Mandarin (M), and reading aloud in English (E). Reading aloud English and Mandarin were performed as baseline tasks (see “Data Analysis” section). Each task included two conditions, which were based on task difficulty, and each condition comprised one block. The eight blocks were administered in a pseudo-random order.

Before each block of the experiment, participants were provided with instructions for the block. The experiment started after the participant pressed a button. At the end of each block, the participants were notified that they had reached the end of the block. The interval between every two blocks was 20 s.

**Figure 1:**
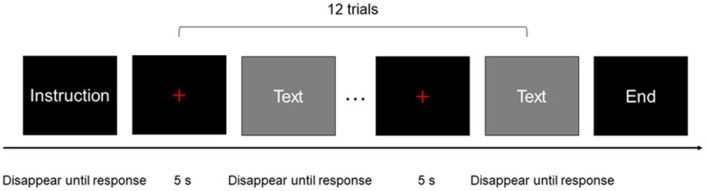
One block of experimental procedure: each rectangle represented a screen of the experimental program presented to participants.

Each block consisted of 12 trials. Each trial included a 5-s pre-stimulus period, during which a red fixation cross was presented in the center of the monitor, followed by a stimulus period. Stimuli did not disappear until the participant finished the trial and pressed a button. A 5-s post-stimulus and recovery period was included after the last stimulus of each block, during which a red fixation cross was displayed in the center of the monitor (Figure 1). A black background was used for all stimuli, the red fixation cross, and instructions. The stimuli and instructions were presented in white text. The size of the red cross was the same as that of the letters/characters used in the stimuli and instructions. All oral outputs were recorded using a digital voice recorder. The recorder was placed near the participants to ensure good sound quality.

The stimuli tasks were programmed using the E-prime software 2.0 (Psychology Software Tools, Sharpsburg, PA). Before the experiment, all participants were trained, to ensure that they were familiar with the experimental procedures and were provided with a warm-up practice consisting of four tasks.

### Data Acquisition

fNIRS data were acquired using an ETG-7100 Optical Topography system (Hitachi Medical Co., Kashiwa, Japan), which used two wavelengths of near-infrared light (695 and 830 nm), with a sampling rate of 10 Hz. The inter-optode distance was 3 cm for each source–detector pair, which facilitated measurements at a 2–3 cm depth from the scalp (Hock et al., 1997).

The probe arrays were mounted on an elastic swimming cap that was worn by each participant, such that the arrays were positioned on the prefrontal lobe and the left and right perisylvian regions. The prefrontal probes measured changes in hemoglobin concentrations using 24 channels in a 4*4 array. Two 3*5 arrays, with 22 channels each, were placed on the bilateral perisylvian regions.

**Figure 2:**
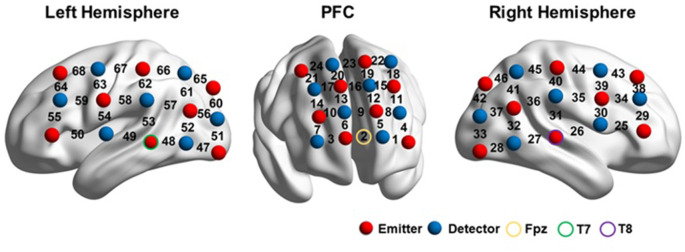
fNIRS setup. The image was visualized with the BrainNet viewer (http://www.nitrc.org/projects/bnv/; Xia et al., 2013).

The probes were positioned according to previously described procedures (Kovelman et al., 2008; Amiri et al., 2014; Perlman et al., 2014; Hong et al., 2015; Fu et al., 2016), to cover the DLPFC, Broca’s area, Wernicke’s area, the inferior parietal lobule, and their homologous regions in the right hemisphere, according to the international 10–20 EEG placement system (Jasper, 1958). The prefrontal lobe was defined as the area above the Fp1-Fp2 line (Kameyama et al., 2004; Hori et al., 2008). Broca’s area was defined as the cross point between T7-Fz and F7-Cz, in the left hemisphere (Kovelman et al., 2008; Schecklmann et al., 2010). Its homolog in the right hemisphere was defined in the cross point between T8-Fz and F8-Cz (ibid). Wernicke’s area and the inferior parietal lobule were defined as the cross point between T7-P3 and C3-P7, in the left hemisphere. Their counterparts in the right hemisphere were defined by the cross point between T8-P4 and C4-P8 (Friederici et al., 1998; Scherer et al., 2012). Therefore, for each participant, the prefrontal probe was positioned such that the middle channel in the lowest row of the probe was placed over Fpz. The bilateral probes were positioned such that the middle detector in the lowest row of optodes was placed over T7 and T8 on the right and left hemispheres, respectively (Figure 2).

After the experiment, the three-dimensional (3D) coordinates of both the sources and detectors on each participant were obtained using a 3D digitizer (PATRIOT, Polhemus, Colchester, Vermont, USA). The average 3D coordinates were calculated and then imported to NIRS_SPM for spatial registration (Singh et al., 2005) to generate a layout of the 46 optodes (Figure 2) and the Montreal Neurological Institute (MNI) coordinates of the 68 channels (Supplementary Material Appendix).

### Data Coding and Scoring

The behavioral data were scored by a panel of five interpreting teachers and scholars, including three native Chinese speakers and two native English speakers. The raters were asked to evaluate reading performance on a 10-point scale, with 10 representing the full score and 0 suggesting a failed reading trail. The criteria for the performance rating included accuracy and fluency. Any inaccurate pronunciation and disfluency resulted in lower scores. Likewise, they were also asked to rate the interpreting trials on a 10-point scale, with 10 representing the full score, indicating a successful interpreting, and 0 indicating a failed interpreting. The criteria for the performance rating included accuracy, fluency, and appropriateness. Any inaccurate expressions, incomplete information, disfluency, or the use of inappropriate expressions or tones resulted in lower scores.

### Data Analysis

A mixed-design analysis of variance (ANOVA, including Interpreting Direction, Interpreting Expertise, and Task Difficulty) was conducted to compare accuracies between groups after the normality test was done. The raw fNIRS data were preprocessed by using NIRS-SPM, Version 4 (Jang et al., 2009; Ye et al., 2009). First, the optical density changes measured by each channel were converted into changes in the concentrations of HbO and HbR, using a modified Beer–Lambert law (Cope and Delpy, 1988). For each participant, the data were preprocessed to remove noises and artifacts (such as head movement and heart rate) using a hemodynamic response function (HRF) filter and a wavelet-minimum description length (MDL) detrending algorithm. All data were valid after preprocessing. Second, a general linear model (GLM) incorporating task effects, a mean, and a linear trend were used to compute parameter estimates. The estimate, β, which represented the levels of brain activation on different channels during different tasks, was obtained. Third, the β values of each interpreting task were normalized against the β values of their corresponding baseline measurements. Two kinds of baseline measurements were used in the present study: (1) FI is normalized using L1 reading, and BI is normalized using L2 reading, in order to control the function of reading; (2) FI is normalized using L2 reading tasks, and BI is normalized using the L1 reading tasks, and both are used to control the function of speaking language aloud. The former was defined as the normalization for reading, the latter was for vocalization. For the normalized β values, mixed-design ANOVA (Interpreting Direction, Interpreting Expertise, and Task Difficulty) was employed for each channel, to explore the differences among the interpreting tasks. A false discovery rate (FDR; *p* < 0.05) was performed for multiple comparisons.

## Results

### Behavioral Results

**Figure 3:**
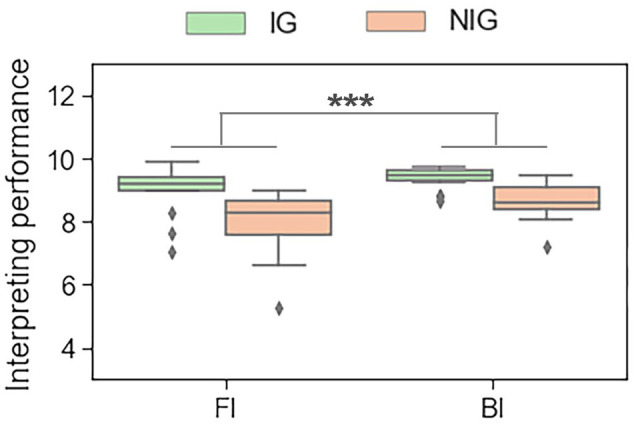
The main effect of interpreting direction in interpreting trials in terms of the behavioral data. ***p < 0.001.

The results showed that, during the interpreting trials, Interpreting Direction had a significant main effect on the performance score (*F*_(1,30)_ = 19.16, *p* < 0.001, partial *η*^2^s = 0.39). All participants performed better in BI tasks than in FI tasks (9.00 ± 0.57 vs. 8.49 ± 1.00; see Figure 3). During the reading trials, Interpreting Direction also had a significant main effect on reading performance (*F*_(1,30)_ = 87.09, *p* < 0.001, partial *η*^2^s = 0.74). Participants had a better performance in reading Mandarin tasks than in reading English tasks (10.00 ± 0.00 vs. 9.80 ± 0.25).

### fNIRS Results

#### Interpreting Direction

**Figure 4:**
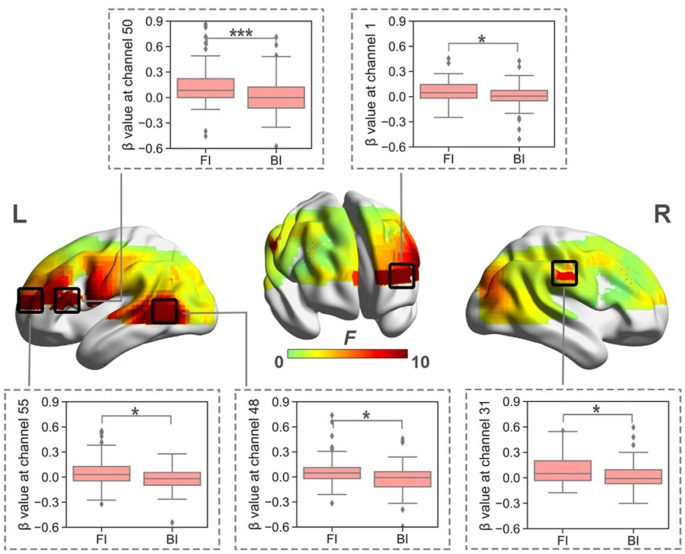
The heat map corresponding to the activated channels impacted by the main effect of interpreting direction in HbO, when normalized for reading; the average β value for such main effect in the activated channels. *p < 0.05; ***p < 0.001. Error bars represent minimum/maximum values. The diamond dots mean extreme values. The shaded area indicates the 95% confidence interval.

When normalized for reading, a significant effect of Interpreting Direction was revealed at a series of channels in HbO (i.e., channel 1, 31, 38, 39, 40, 42, 48, 49, 50, 55, 61, and 63; *F*s > 4.28, *p*s < 0.05, partial *η*^2^s > 0.12) and HbR (i.e., channel 12, 13, 15, 16, 18, 20, 23, 26, 30, 46, 56, 61, 65, 66; *F*_s_ > 4.03, *p*_s_ < 0.05, partial *η*^2^s > 0.11) before FDR correction. However, after FDR correction, significant effects only remained in HbO for channels 1, 31, 48, 50, and 55 (Figure 4). These channels were approximately located at left frontopolar area (BA 10), bilateral temporal cortex (BA 21, 22), and left Broca’s area (BA 45).

When normalized for vocalization, the results indicated that Interpreting Direction had no significant effect on brain activation in HbO (*F*_s_ < 2.81, *p*_s_ > 0.09; partial *η*^2^s < 0.09) or HbR (*F*_s_ < 3.49, *p*_s_ > 0.07; partial *η*^2^s < 0.11) before FDR correction.

#### Interpreting Direction and Interpreting Expertise

**Figure 5:**
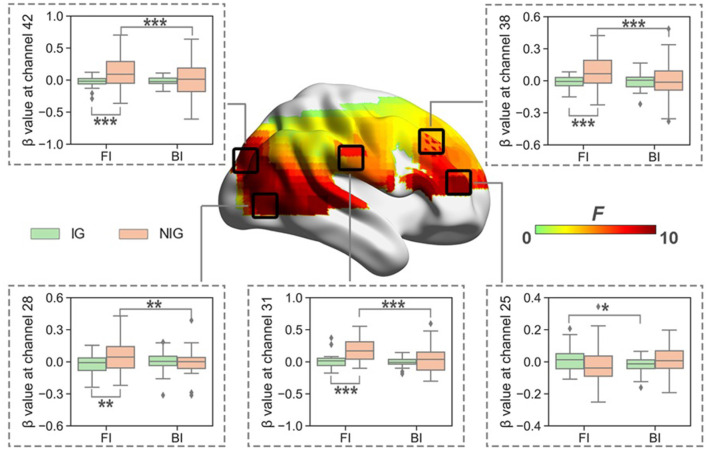
The heat map corresponding to the activated channels impacted by the interaction between interpreting direction and interpreting expertise in HbO, when normalized for reading; the average β value for such interaction in the activated channels. *p < 0.05; **p < 0.01; ***p < 0.001. Error bars represent minimum/maximum values. The diamond dots mean extreme values. The shaded area indicates the 95% confidence interval.

When normalized for reading, the results showed significant interaction between Interpreting Direction and Interpreting Expertise in HbO (i.e., channel 1, 3, 25, 26, 28, 31, 32, 38, 42, 48, 49, 55, 59, 61, and 63; *F*s > 5.79, *p*_s_ < 0.03, partial *η*^2^s > 0.16) and HbR (i.e., channel 16, 17, 20, 46, and 66; *F*_s_ > 4.02, *p*_s_ < 0.05, partial *η*^2^s > 0.11). After FDR correction, channels 25, 28, 31, 38, and 42 of HbO remained (Figure 5). These channels roughly covered right DLPFC (BA 9), right visual area 3 (V3, BA 19), right superior temporal gyrus (STG, BA22), and right Broca’s area (BA 45).

The simple effect test revealed that at channel 25, which belonged to the right Broca’s area, IG had higher activation in the FI condition than that in the BI condition (*t* = 2.10, *p* = 0.04, Cohen’s *d* = 0.52; 0.01 ± 0.07 vs. −0.02 ± 0.05).

At channels 28, 31, 38, and 42, which roughly covered right DLPFC, right visual area 3, and right STG, NIG showed stronger brain activation in the FI condition than that in the BI condition (*t*_s_ > 3.17, *p*_s_ < 0.01, Cohen’s *d*_s_ > 0.79); and NIG activated more in the FI condition than IG (*t*_s_ > 3.13, *p*_s_ < 0.01, Cohen’s *d*_s_ > 0.70).

**Figure 6:**
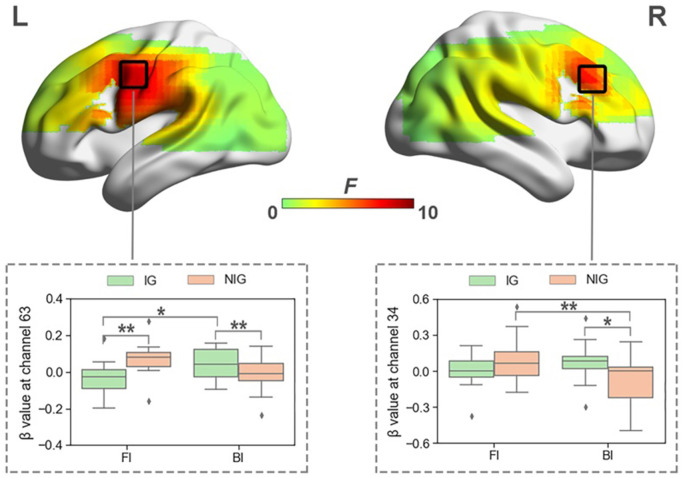
The heat map corresponding to the activated channels impacted by the interaction between interpreting direction and interpreting expertise in HbO, when normalized for vocalization; the average β value for such interaction in the activated channels. *p < 0.05; **p < 0.01. Error bars represent minimum/maximum values. The diamond dots mean extreme values. The shaded area indicates the 95% confidence interval.

When normalized for vocalization, a significant interaction was also found between Interpreting Direction and Interpreting Expertise in HbO and HbR before FDR correction. The activated channels in HbO included channels 34, 38, 39, 50, 54, 58, 59, and 63 (*F*_s_ > 4.04, *p*_s_ < 0.05, partial *η*^2^s > 0.11). The results of HbR included channels 10, 14, 25, 29, 30, 34, 35, 36, and 44 (*F*_s_ > 3.91, *p*_s_ < 0.05, partial *η*^2^s > 0.12). However, after FDR correction, only channels 34 and 63 in HbO remained (Figure 6), no channel in HbR survived. Channel 34 was approximately located at the right Broca’s area (BA 44), and channel 63 was roughly located at the Premotor and Supplementary Motor Cortex (BA 6).

The simple effect test revealed that at channel 34, which was approximately located at right Broca’s area, IG activated more in the BI condition than NIG (*t* = 2.41, *p* = 0.02, Cohen’s *d* = 0.55; 0.07 ± 0.16 vs. −0.10 ± 0.25); participants in NIG showed larger brain activity in the FI condition than the BI condition (*t* = 2.92, *p* = 0.007, Cohen’s *d* = 0.73; 0.08 ± 0.20 vs. −0.01 ± 0.13).

At channel 63, roughly located at Premotor and Supplementary Motor Cortex, IG activated more than NIG in the BI condition (*t* = −2.04, *p* = 0.04, Cohen’s *d* = 0.56; −0.01 ± 0.10 vs. 0.08 ± 0.19), whereas NIG produced more intensified brain activity in the FI condition (*t* = 2.52, *p* = 0.01, Cohen’s *d* = 0.70; −0.04 ± 0.10 vs. 0.07 ± 0.09); participants in IG exhibited higher activity in the BI condition than the FI condition (*t* = −2.50, *p* = 0.02, Cohen’s *d* = 0.63; 0.08 ± 0.19 vs. −0.04 ± 0.10).

#### Interpreting Direction, Interpreting Expertise, and Task Difficulty

When normalized for reading, no significant interaction was identified among Interpreting Direction, Interpreting Expertise, and Task Difficulty in HbO or HbR before FDR correction (HbO: *F*_s_ < 5.50, *p*_s_ > 0.02, partial *η*^2^s < 0.16; HbR: *F*_s_ < 2.73, *p*_s_ > 0.09, partial *η*^2^s < 0.09).

When normalized for vocalization, such interaction was nonsignificant in HbO before FDR correction (*F*_s_ < 3.44, *p*_s_ > 0.07; partial *η*^2^s < 0.11). Although such interaction effect was significant in HbR at channel 39 and 43 before FDR correction (*F*_s_ > 5.58, *p*_s_ < 0.03, partial *η*^2^s > 0.15), no significant result revealed after the correction.

These results might suggest the relationship between Interpreting Direction and Interpreting Expertise may be independent of task difficulty, in terms of the cognitive processing required during interpreting.

## Discussion

The present study was designed to investigate the neural mechanisms underlying the directionality in English/Mandarin sight translation and how it correlates with interpreting expertise and task difficulty. The findings shed light not only on directionality and expertise but also reveal the relevance of fNIRS for a similar investigation. Future research examining this issue is likely to provide equally interesting findings by studying other groups or utilizing other methodologies. In the following section, we summarize and discuss our findings.

### Directionality

When the data were normalized for reading, FI resulted in more pronounced brain activation for all participants, indicating that more cognitive effort was required when interpreting in this direction compared with BI. This result corroborates the findings of Klein et al. (1995), Tommola et al. (2000), He et al. (2017), and Jost et al. (2018), who found more intensified brain activation during FI than during BI. This result collaborates with the *inhibitory control* model. It is suggested that sight translating from L1 to L2 requires cognitive effort to suppress L1 lemmas, whilst sight translating from L2 to L1 requires effort to suppress L2 lemmas. Since L1 lemmas is more active than L2 lemmas, it requires more effort to suppress L1 lemmas than to suppress L2 lemmas. This seems to explain why FI requires more effort than BI in general.

The directionality effect caused significantly intensified brain activation in the left frontopolar area, bilateral temporal cortex, and left Broca’s area, which might indicate that interpreting from L1 into L2 required more cognitive efforts in these brain regions.

The frontopolar area has been suggested to be involved in the maintenance of a previously running task, in a suspended state, for subsequent retrieval and execution while completing ongoing tasks. Koechlin and Hyafil (2007) further proposed that this area may be related to the performance of a domain-general function when scheduling multiple tasks that are engaged simultaneously during complex mental activities. This area has also been found to be involved in episodic memory, working memory, and coordination among multiple tasks (Gilbert et al., 2006). The directionality effect in this area may indicate that FI requires more executive control than BI, such as multi-task coordination.

Left Broca’s area has been associated with syntactic processing (Embick et al., 2000; Friederici and Kotz, 2003) as well as semantics and phonological processing (Tate et al., 2014). In addition to its linguistic functions, studies have suggested that it may also be involved in cognitive functions, such as lexical retrieval, cognitive control, and verbal working memory during language processing (Tettamanti and Weniger, 2006; Tate et al., 2014). The directionality effect observed for this area might indicate that more effort is required to perform language production and executive functions, such as lexical retrieval and verbal working memory, during FI than during BI. This result corroborates the findings reported by Tommola et al. (2000) and He et al. (2017), whose studies also revealed more pronounced brain activation in Broca’s area elicited by FI than that by BI.

The temporal cortex has been suggested by Sato et al. (1999) to play a role in memory storage and retrieval, which was also supported by Menenti et al. (2012), whose study indicated the critical role played by the middle temporal gyrus in the retrieval of lexical-syntactic information during language production. Furthermore, previous studies have found that the activation of this brain region was associated with semantic processing, including the integration of semantic information with previous sentence information (Franzmeier et al., 2012) and the selection of subordinate meanings (Whitney et al., 2011; Hoffman et al., 2012). The stronger activation of this area during FI might suggest that more effort is necessary for semantic processing during language production in this direction, including accessing the lexical-syntactic information for L2 production. This result is consistent with the EEG study performed by Kurz (1995), which demonstrated that relative to the resting state, active interpreting caused more pronounced left temporal activation for FI.

The more intensified activation in the left frontopolar area, left Broca’s area, and the left temporal cortex might also suggest FI requires more effort for semantic control. Semantic control is the ability to selectively access and manipulate meaningful information according to context demands (Jackson, 2021). According to Ralph et al. (2017), brain activity within the network for semantic representation must be controlled to ensure that the system produces representations and inferences which are appropriate for the immediate tasks. The semantic control network is intended to support working memory as well as executive representations which encode information about the temporal and situational context relevant to the immediate task (ibid). In well-rehearsed tasks in which relevant information is strongly encoded, the representation network needs little input from semantic control to generate the right response. Yet for tasks requiring retrieval of weakly encoded information and suppression of over-practiced responses, more input from the control network is needed (ibid). Neuroimaging and patient studies (i.e., Whitney et al., 2011; Ralph et al., 2017; Jackson, 2021) have suggested the semantic control network involves the left prefrontal and posterior middle temporal regions. In the present study, FI activating such brain areas may suggest more effort is required for generating representations and inferences that are suited to sight translating from L1 to L2, for suppressing strong associates in L1 of a given concept, and for encoding relevant information in L2.

The results of the present study are also consistent with those of Kurz (1995), who also identified the involvement of the right hemisphere in FI. However, when the data were normalized for vocalization, no main effect of interpreting direction was found in terms of the brain data for all participants.

### Directionality Modulated by Interpreting Expertise

When normalized for reading, our findings revealed a significant interaction between interpreting direction and interpreting expertise, which indicated that interpreting direction had different effects on cognitive processing that depend on the level of interpreting expertise of the experimental participants.

For IG, interpreting direction had a significant main effect in the right Broca’s area, and FI produced more cognitive load in this brain region. For NIG, interpreting direction had a significant main effect in the right DLPFC, the right STG, and the right V3, with FI producing more cognitive load in these brain regions.

The observed group difference might indicate that interpreting experience and training may cause changes in the neural substrates associated with interpreting in the right hemisphere, including the inferior frontal cortex, the dorsolateral prefrontal cortex, the superior temporal gyrus, and V3. These brain regions have been suggested to play roles in cognitive inhibition, working memory, language switching (the ability to selectively activate one language while suppressing others), and language processing activities. This finding is similar to the findings reported by studies that have compared professional interpreters with non-interpreters, even when matched for L2 proficiency, which revealed that interpreting expertise might modulate executive control (Ibanez et al., 2010; Yudes et al., 2011).

The intensified activation of the right Broca’s area was found in the IG. Aron et al. (2004) reported that converging evidence has indicated that the right inferior frontal gyrus (including BA 44/45) plays a key role in the inhibitory processes that underlie language switching. According to their studies, the right inferior frontal gyrus, which is the homolog of Broca’s area in the right hemisphere, might be closely related to cognitive inhibition, which is a component of executive control. They further defined cognitive inhibition as the voluntary blocking of interfering memory during retrieval and cognitive suppression of inappropriate responses (ibid). Therefore, the intensified activation of this area during FI that was observed for IG is likely due to the inhibition of non-target translation options, in addition to interfering with memory retrieval.

The activation of the dorsolateral prefrontal cortex in the right hemisphere was observed for NIG. It is well-known for being involved in executive functions, including planning, abstract reasoning, working memory, and attention. Also, it is involved in task monitoring, especially during unfamiliar or attention-demanding tasks (Kovelman et al., 2008), and language switching in bilinguals (Price et al., 1999; Hernandez et al., 2001). Similarly to the frontopolar prefrontal cortex, it is also important for holding several pieces of transitory information in the mind before the information can be processed (Nelson and Luciana, 2001). Studies examining memory and language have confirmed the presence of strong empirical connections between the dorsolateral prefrontal cortex and working memory (Gabrieli, 1998; Baddeley, 2000). The dorsolateral prefrontal cortex is thought to be involved in controlling working memory and attentional resources (Fuster, 2008; Balconi, 2013). Jasińska and Petitto (2014) attributed the activation of the prefrontal cortex, including both the rostrolateral prefrontal cortex and the dorsolateral prefrontal cortex, to the employment of cognitive processes, such as working memory, attention, and reasoning, and claimed these were essential processes for switching between languages. This finding was supported by those reported by Mücke et al. (2018). The right dorsolateral prefrontal cortex has also been suggested to be involved in tasks that require visual working memory, according to Smith et al. (1996), supported by Kovelman et al. (2008).

The right dorsolateral prefrontal cortex is suggested to play a critical role in the cognitive processing required for interpreting tasks, such as visual working memory, switching between two languages, and controlling attentional resources. The more pronounced brain activation of this area observed during FI for NIG might be explained by the cognitive efforts required to perform executive functions during an interpreting task. Owing to a lack of experience and practice, non-interpreter bilinguals were not familiar with the process of producing target text orally while comprehending the source text. The whole process, which requires multi-tasking coordination, switching between two languages, working memory, and attentional resources, imposed more cognitive challenges on NIG than on IG, who have internalized this process so that it occurs automatically, without cognitive effort. According to Göpferich et al. (2011), expertise development is associated with increased automatic processing and reduced reliance on working memory. As a result, experienced interpreters might be less vulnerable than non-interpreter bilinguals to the impacts of resource competition for working memory (Lacruz, 2017).

For NIG, FI also activated the right STG and the right V3, which are suggested to be responsible for language processing activities and the cognitive processing of visual stimuli, respectively. The homologous area of STG in the non-dominant hemisphere is reported to be involved in language processing. The research conducted by Harpaz et al. (2009) suggested that this area plays a role when processing and determining the secondary meaning of words. Also, studies (e.g., Poeppel et al., 2008; Newman et al., 2010) have suggested the right STG might be recruited during language activities. This may well explain why these areas were activated more intensively during FI for NIG, as more effort might be required for language processing in L2 for this group. This result also indicated that the untrained bilinguals suffered from cognitive loads as the result of sight translation, which likely represents an interpreting modality that they were not familiar with.

When the fNIRS data were normalized for vocalization, we also found a significant interaction effect between interpreting direction and interpreting expertise. For IG, interpreting direction had a significant effect in the premotor and supplementary motor cortex in the left hemisphere, with BI producing more cognitive load in this brain region. The premotor and supplementary motor cortex has been included in the language network as a region that is linked to the inferior frontal language area (Dick et al., 2014) and seems to be primarily involved in speech motor control. There is increasing evidence showing that this area is also involved in other language functions such as verbal working memory and predictive top-down mechanisms during speech perception (Hertrich et al., 2016). The activation of this brain region might suggest its involvement in English-Mandarin interpreting for more experienced interpreters. Interestingly, for NIG, interpreting direction had a significant effect in the right Broca’s area, with FI producing more cognitive load in this brain region. This may also be due to the inhibition of non-target translation options, apart from the interference with memory retrieval.

The neuroimaging results were not consistent with the behavioral results, in terms of the main effect of interpreting direction as well as the interaction between interpreting direction and experience. This corroborates the findings of an event-related potential study, performed by Elmer et al. (2010), who found intergroup consistency in behavioral results and enlarged N400 responses among professional interpreters. Previous studies have not yet identified any simple one-to-one correspondence between behavioral performance and neural activation, which is common in language processing studies, where incongruent language groups can show identical behavioral performances and incongruent neural profiles (Kovelman et al., 2008).

## Conclusions

This study aimed to examine the directionality effect in Mandarin/English interpreting, from a neurocognitive perspective, through the employment of fNIRS methodology. Our findings suggested that when normalized for reading, interpreting direction had an effect on brain activation, with FI eliciting more intensified brain activation than BI in the left frontopolar area, bilateral temporal cortex, and left Broca’s area. The more pronounced activation of these areas might suggest that during FI, more effort is required to perform multi-task coordination, lexical and lexical-syntactic information retrieval during speech production, and semantic information integration and control, than during BI.

Additionally, directionality caused incongruent activation patterns that were modulated by interpreting expertise. FI activated right Broca’s area in IG, whereas right DLPFC, right STG, and right V3 were activated in NIG. This finding seems to suggest that for interpreters, FI requires more effort associated with executive function, related to cognitive inhibition, the voluntary blocking of interfering memory during retrieval, and the cognitive suppression of inappropriate response, whereas non-interpreter bilinguals experienced higher cognitive loads associated with the executive functions necessary for multi-task coordination, language switching, attentional resource allocation, and language processing.

When normalized for vocalization, though no main effect of interpreting direction was identified, we did find an interaction effect between interpreting direction and interpreting expertise. For interpreters, backward interpreting activated the left premotor and supplementary motor cortex, whilst for non-interpreter bilinguals, forward interpreting activated the right Broca’s area. This again reveals the different effects of interpreting direction on cognitive processing that depend on the level of interpreting expertise. The results corroborate the *inhibitory control* model in that for unbalanced bilinguals translating from L1 to L2 is more cognitively loaded. Also, the findings suggest the important role the premotor and supplementary motor cortex, as well as the right Broca’s area, have played in English/Mandarin interpreting.

In this study, fNIRS was a highly beneficial technique to uncover the cognitive processing and neural mechanisms under natural working conditions in real-world circumstances. However, the conclusions we could draw from fNIRS are limited by its resolution and thus are considered tentative. The findings could also be limited given that we had not included English (L1)/Mandarin (L2) bilinguals performing the same tasks, thus follow-up studies should be conducted to further investigate this issue.

## Data Availability Statement

The raw data supporting the conclusions of this article will be made available by the authors, without undue reservation.

## Ethics Statement

The protocol was reviewed and approved by the Committee on Human Research Protection of East China Normal University (HR 094-2018). All participants signed informed consent forms prior to the experiment.

## Author Contributions

YHe wrote the manuscript. YY analyzed the data. YinHu, DL, and YiHu reviewed and revised the manuscript. All authors contributed to the article and approved the submitted version.

## Conflict of Interest

The authors declare that the research was conducted in the absence of any commercial or financial relationships that could be construed as a potential conflict of interest.

## Publisher’s Note

All claims expressed in this article are solely those of the authors and do not necessarily represent those of their affiliated organizations, or those of the publisher, the editors and the reviewers. Any product that may be evaluated in this article, or claim that may be made by its manufacturer, is not guaranteed or endorsed by the publisher.
