# Supplementary material for: Optical Mapping of Brain Activity Underlying Directionality and Its Modulation by Expertise in Mandarin/English Interpreting
Source: Front Hum Neurosci. 2021 Aug 6;15:649578. doi: 10.3389/fnhum.2021.649578 (PMC8377287; doi:10.3389/fnhum.2021.649578)
Supplement: Supplementary file 1 [file Data_Sheet_1.docx]

**Appendices**

**Appendix 1 Comparability of Mandarin and English sentences**

|  | LC sentences | | HC sentences | |
| --- | --- | --- | --- | --- |
|  | Mandarin | English | Mandarin | English |

| Average word count | 10.13 | 10.08 | 10 | 10 |
| --- | --- | --- | --- | --- |
| Frequent word percentage (%)  within 1-1000 interval  1001-2000 interval  2001-3000 interval  3001-4000 interval  4001-5000 interval  above 5000 interval | 89.88  5.56  2.92  0.76  0.46  0.42 | 90.07  4.47  3.38  0.83  0.42  0.83 | 69.46  8.36  4.55  0.83  2.64  14.15 | 68.60  8.40  4.14  1.67  2.93  14.26 |
| Average notional word density (%) | 81.19 | 78.63 | 78.95 | 78.09 |
| Average pronoun density (%) | 10.33 | 11.61 | 10.51 | 11.67 |
| Average translatability value | 2.76 | 2.64 | 3.76 | 3.80 |

**Appendix 2 Distinction between LC and HC sentences in both languages**

|  | Mandarin sentences | English sentences |
| --- | --- | --- |
|  | *p*-value | *p*-value |

| Frequent word percentage (%)  within 1-1000 interval  1001-2000 interval  2001-3000 interval  3001-4000 interval  4001-5000 interval  above 5000 interval | 0.00  0.19  0.29  0.93  0.04  0.00 | 0.00  0.04  0.65  0.47  0.02  0.00 |
| --- | --- | --- |
| Average translatability value | 0.00 | 0.00 |

**Appendix 3 The 3D MNI Coordinates of the 68 Channels and the Corresponding Brain Area**

| Channels | MNI coordinates | | | Hemisphere | Brodmann area | Area for localization |
| --- | --- | --- | --- | --- | --- | --- |
|  | x | y | z |  |  |  |
| 01 | -23 | 70 | 13 | L | 10 | Frontopolar area |
| 02 | -1 | 68 | 14 | L | 10 | Frontopolar area |
| 03 | 22 | 71 | 15 | R | 10 | Frontopolar area |
| 04 | -32 | 62 | 18 | L | 10 | Frontopolar area |
| 05 | -13 | 69 | 22 | L | 10 | Frontopolar area |
| 06 | 14 | 68 | 26 | R | 10 | Frontopolar area |
| 07 | 32 | 63 | 22 | R | 10 | Frontopolar area |
| 08 | -21 | 62 | 30 | L | 10 | Frontopolar area |
| 09 | 0 | 61 | 33 | \ | 10 | Frontopolar area |
| 10 | 20 | 62 | 33 | R | 10 | Frontopolar area |
| 11 | -31 | 50 | 36 | L | 9 | Dorsolateral prefrontal cortex |
| 12 | -11 | 58 | 42 | L | 9 | Dorsolateral prefrontal cortex |
| 13 | 13 | 58 | 41 | R | 9 | Dorsolateral prefrontal cortex |
| 14 | 30 | 51 | 38 | R | 9 | Dorsolateral prefrontal cortex |
| 15 | -18 | 48 | 47 | L | 9 | Dorsolateral prefrontal cortex |
| 16 | 2 | 49 | 47 | R | 8 | Dorsolateral prefrontal cortex |
| 17 | 20 | 48 | 49 | R | 8 | Dorsolateral prefrontal cortex |
| 18 | -32 | 31 | 53 | L | 8 | Dorsolateral prefrontal cortex |
| 19 | -11 | 40 | 57 | L | 8 | Frontal eye fields |
| 20 | 12 | 41 | 57 | R | 8 | Frontal eye fields |
| 21 | 29 | 33 | 55 | R | 8 | Frontal eye fields |
| 22 | -20 | 26 | 63 | L | 8 | Frontal eye fields |
| 23 | 0 | 29 | 60 | \ | 8 | Frontal eye fields |
| 24 | 20 | 26 | 63 | R | 8 | Frontal eye fields |
| 25 | 61 | 25 | 9 | R | 45 | pars triangularis Broca's area |
| 26 | 72 | -12 | 4 | R | 22 | Superior Temporal Gyrus |
| 27 | 72 | -44 | 1 | R | 22 | Superior Temporal Gyrus |
| 28 | 56 | -74 | -5 | R | 19 | Visual area V3 |
| 29 | 55 | 39 | 19 | R | 45 | pars triangularis Broca's area |
| 30 | 67 | 5 | 21 | R | 6 | Premotor and Supplementary Motor Cortex |
| 31 | 71 | -27 | 18 | R | 22 | Superior Temporal Gyrus |
| 32 | 64 | -60 | 13 | R | 37 | Fusiform gyrus |
| 33 | 49 | -85 | 5 | R | 19 | Visual area V3 |
| 34 | 58 | 23 | 30 | R | 44 | Broca's area |
| 35 | 69 | -9 | 32 | R | 43 | Subcentral area |
| 36 | 69 | -42 | 29 | R | 40 | Supramarginal gyrus part of Wernicke's area |
| 37 | 57 | -71 | 24 | R | 39 | Angular gyrus |
| 38 | 47 | 36 | 39 | R | 9 | Dorsolateral prefrontal cortex |
| 39 | 59 | 5 | 43 | R | 6 | Premotor and Supplementary Motor Cortex |
| 40 | 67 | -25 | 45 | R | 1 | Primary Somatosensory Cortex |
| 41 | 61 | -58 | 39 | R | 39 | Angular gyrus |
| 42 | 44 | -83 | 29 | R | 19 | Visual area V3 |
| 43 | 47 | 22 | 51 | R | 9 | Dorsolateral prefrontal cortex |
| 44 | 57 | -9 | 53 | R | 4 | Primary Motor Cortex |
| 45 | 61 | -40 | 53 | R | 40 | Supramarginal gyrus part of Wernicke's area |
| 46 | 48 | -70 | 47 | R | 39 | Angular gyrus |
| 47 | -50 | -83 | -6 | L | 19 | Visual area V3 |
| 48 | -66 | -55 | -2 | L | 37 | Fusiform gyrus |
| 49 | -71 | -24 | 0 | L | 21 | Middle Temporal gyrus |
| 50 | -60 | 14 | 6 | L | 48 | Retrosubicular area |
| 51 | -40 | -95 | 1 | L | 18 | Visual Association Cortex (V2) |
| 52 | -58 | -72 | 8 | L | 19 | Visual area V3 |
| 53 | -70 | -41 | 11 | L | 22 | Superior Temporal Gyrus |
| 54 | -67 | -5 | 18 | L | 43 | Subcentral area |
| 55 | -56 | 32 | 14 | L | 45 | pars triangularis Broca's area |
| 56 | -50 | -82 | 19 | L | 19 | Visual area V3 |
| 57 | -65 | -55 | 25 | L | 22 | Superior Temporal Gyrus |
| 58 | -68 | -21 | 29 | L | 2 | Primary Somatosensory Cortex |
| 59 | -61 | 11 | 27 | L | 44 | Broca's area |
| 60 | -36 | -91 | 26 | L | 18 | Visual Association Cortex (V2) |
| 61 | -54 | -70 | 37 | L | 39 | Angular gyrus |
| 62 | -65 | -39 | 41 | L | 40 | Supramarginal gyrus part of Wernicke's area |
| 63 | -61 | -4 | 40 | L | 6 | Premotor and Supplementary Motor Cortex |
| 64 | -49 | 28 | 38 | L | 44 | Broca's area |
| 65 | -39 | -80 | 44 | L | 19 | Visual area V3 |
| 66 | -56 | -50 | 52 | L | 40 | Supramarginal gyrus part of Wernicke's area |
| 67 | -58 | -19 | 53 | L | 3 | Primary Somatosensory Cortex |
| 68 | -47 | 16 | 51 | L | 9 | Dorsolateral prefrontal cortex |
